# Acknowledgment of *Ad Hoc* Reviewers

**DOI:** 10.1128/mSphere.00358-16

**Published:** 2016-12-28

**Authors:** Michael J. Imperiale

**Affiliations:** University of Michigan, Ann Arbor, Michigan, USA

## EDITORIAL

As we reach the end of our first full year of publishing cutting-edge research in the microbial sciences, we would like to offer our sincere thanks to the many individuals who have acted as referees of the manuscripts that we have handled since our birth in late 2015. They have helped us achieve our goal of delivering rapid, fair decisions to our authors.

Mark D. Adams
Evelien M. Adriaenssens
Jesus Aguirre
Lee-Ann H. Allen
Lysangela Ronalte Alves
Antoine Andremont
Largus T. Angenent
Natalie G. Anosova
Charles S. Apperson
Rajendra Apte
David M. Aronoff
Gustavo Arrizabalaga
Marina S. Ascunce
Walid Azab
Alexandre M. Bailao
Niaz Banaei
Jeffrey A. Banas
E. Magda Barbu
Stephen J. Barenkamp
Peter Barlow
Susan Baserga
Jesiane Batista
John Battista
Margaret E. Bauer
Anthony D. Baughn
Adi Behar
George A. Belov
William H. Benjamin
Ivan Berg
Teresa M. Bergholz
Kirk Bergstrom
Luiz E. Bermudez
Emily Bernstein
Corina M. Berón
Xavier Bertrand
Rodrigo Carvalho Bicalho
Krystyna Bienkowska-Szewczyk
Elisabeth M. Bik
Thomas Binz
Jill R. Blankenship
David S. Blehert
Linda K. Bockenstedt
Carolyn H. Bohach
Jeffrey L. Bose
Helena Ingrid Boshoff
Catharine M. Bosio
Ethna Fidelma Boyd
Jon P. Boyle
Salvatore Bozzaro
Curtis R. Brandt
Morten Kielland Brandt
Gerhard H. Braus
Erhard Bremer
Heike Brötz-Oesterhelt
Alex Brown
Alistair J. P. Brown
Barbara A. Brown-Elliott
Harald Bruessow
Sascha Brunke
Christopher Brian Buck
Tucker Burch
Vincent Burrus
Peter Bütikofer
Noah Butler
Yolanda Cajal
Douglas R. Call
Ana M. Calvo
Andrew Camilli
Alessandra Carattoli
Heather Carleton
Vern B. Carruthers
Dee A. Carter
James E. Cassat
Vincent Cattoir
Lindsay J. Caverly
TingFung Chan
Kartik Chandran
Theresa Chang
Vishnu Chaturvedi
Neeraj Chauhan
Sheng Chen
Melissa Rae Christopherson
Ousmane Cisse
Thomas Clavel
Steven Clegg
Daniel Lee Clemens
Kenneth D. Clinkenbeard
Luca Cocolin
Noah D. Cohen
Eric S. Cole
Luis Comolli
Brian P. Conlon
Patricia S. Conville
Cynthia Nau Cornelissen
Michael Cox
Robert A. Cramer
Alison K. Criss
Liwang Cui
Longzhu Cui
Etienne Dague
Holger Daims
John Daiss
David Allan Brett Dance
Andre Dascal
Philip Davidson
Scott C. Dawson
Maria De Angelis
Valérie de Crécy-Lagard
Miranda De Graaf
Kirk W. Deitsch
Boudewijn L. De Jonge
Maurizio Del Poeta
John Demain-Sauer
Jane C. Deng
Eric Y. Denkers
Joseph L. DeRisi
Lalitagauri Deshpande
Diego De Stefano
Ramon Diaz-Orejas
Manlio Di Cristina
Andreas Diepold
Stephen P. Diggle
Dirk P. Dittmer
Maureen J. Donlin
Adam Driks
Aurore Dubuffet
Edward G. Dudley
Melissa B. Duhaime
Eileen M. Dunne
Gary M. Dunny
Rachel J. Dutton
Anne Eady
Jeffrey L. Ebersole
Gabriela Echaniz-Aviles
Mira Edgerton
Elizabeth A. Edwards
Armin Ehrenreich
Patrick Eichenberger
Joel C. Eissenberg
Marie A. Elliot
Andrea Endimiani
Tobias Erb
Aaron Conrad Ericsson
M. Z. Fan
Nicolas Fasel
Herman W. Favoreel
Mariet C. Feltkamp
J. Christopher Fenno
Noah Fierer
Scott G. Filler
Jeffrey S. Fillingham
Daniel P. Flaherty
Katrina T. Forest
Jarrod R. Fortwendel
Fiona Fouhy
James A. Fraser
Pina M. Fratamico
Stephen J. Free
David N. Frick
Michael Y. Galperin
Thomas Geisbert
Sudeshna Ghosh
Tommaso Giani
Deanna Gibson
Scott Michael Gifford
Jason Gigley
Britt A. Glaunsinger
Eva Gluenz
Mark Gomelsky
Sebastian Gornik
Sven B. Gould
Benjamin Gourion
Revathi Govind
Steven T. Gregory
Matt J. Griffin
Trudy H. Grossman
Bradley Groveman
Marc-Jan Gubbels
Sarah-Jane Haig
Stephen L. Hajduk
Pamela R. Hall
Kim Hare
Miranda Hart
Erica Hartmann
Leiv Sigve Håvarstein
Nicholas S. Heaton
Christine Heilmann
David E. Heinrichs
Regine Hengge
Michael Hensel
Deborah A. Hogan
Stefan Hohmann
Saul M. Honigberg
Stacy M. Horner
Daniel K. Howe
Joseph Hoyt
Huan Huang
Alexander Idnurm
Frederick Immermann
Lakshminarayan M. Iyer
Robert Jackson
Mohamed A. Jamal
Babak Javid
Victoria Jeffers
James R. Johnson
Christopher J. Jones
Susan Joyce
Praveen Rao Juvvadi
David Kadosh
Bjorn Kafsack
Lindsay Kalan
Alex J. Kallen
Jeremy Phillip Kamil
James A. Karlowsky
Sophia Kathariou
Thomas E. Kehl-Fie
Gary Ketner
Morten C. Kielland-Brandt
Mogens Kilian
Byung S. Kim
Paul R. Kinchington
Robert A. Kingsley
Girish Soorappa Kirimanjeswara
Lawrence A. Klobutcher
Donald P. Knowles
Kouacou V. Konan
James B. Konopka
Rolf Krause
James W. Kronstad
Laurie T. Krug
Adam C. Krull
Brenda A. Kwambana-Adams
Thomas Lane
Felix Lankester
Alison Laufer Halpin
Adam S. Lauring
Samuel A. Lee
Soo Chan Lee
Emil P. Lesho
Cammie Lesser
Xiaorong Lin
Scott Lindner
John J. LiPuma
Per O. Ljungdahl
Fabián Lorenzo-Díaz
Julius Lukes
Andrew J. MacAdam
Jean-Yves Madec
Paul Magwene
Bernardo Alfredo Mainou
Hemanta K. Majumder
Nicholas J. Mantis
Luis Martínez
Jean-Christophe Marvaud
Christopher Mason
Jyl S. Matson
John K. McCormick
Rebecca McCulley
Daniel McDonald
Andrew McDowell
Alastair McEwan
Bradford S. McGwire
Kevin S. McIver
Marc Meneghini
Matthew Mikoleit
Alita A. Miller
Raul Miranda-CasoLuengo
Chad Mire
Nathaniel John Moorman
Beatriz M. Moreira
Ignacio Moriyon
Meredith Teilhet Morris
Uffe Hasbro Mortensen
Elke Mühlberger
Carol A. Munro
Vincent J. Munster
Eain A. Murphy
Kenan C. Murphy
Michael Murphy
Mustapha M. Mustapha
Valakunja Nagaraja
Martha I. Nelson
Glen Nemerow
Gilles Nevez
Dao Nguyen
Max L. Nibert
Connie Nichols
Wright W. Nichols
Kirsten Nielsen
Morten Nielsen
James P. O’Gara
Erica Ollmann Saphire
Michael Olson
Klaus Osterrieder
Arthur Ouwehand
Mark G. Papich
Mark S. Parcells
Daniel Paredes-Sabja
Alexander R. Paredez
Heesoo Park
Colin R. Parrish
Cheryl L. Patten
Shelley M. Payne
Victoria Pederick
Vladimir Pelicic
William Penwell
Daniel R. Perez
Julie K. Pfeiffer
Gregory J. Phillips
Renata Cristina Picão
Johann Pitout
Michael R. Popoff
Andrew Preston
Michael J. Pucci
Elinor deLancey Pulcini
Huan Qiu
Cheryl L. Quinn
Robert G. Quivey
Bethany A. Rader
Michael Rappe
Rino Rappuoli
David A. Rasko
Philip N. Rather
Douglas Scott Reed
Michael Reese
Joice N. Reis
Michelle Lynne Reniere
Federico Rey
Todd B. Reynolds
Ruth E. Richardson
Lee W. Riley
Roy Robins-Browne
Jesús Rodríguez-Baño
David Rogers
Marat R. Sadykov
Jason William Sahl
Wilmara Salgado-Pabón
Philippe J. Sansonetti
Irene Santamarta
Renato Lima Santos
Karla J. F. Satchell
Khalid Sayood
Hubert Schaller
Luis M. Schang
Sergio Schenkman
Achim Schnaufer
Jared Schrader
Stacey Schultz-Cherry
Ingrid L. Scully
Lynne Sehulster
David A. Sela
Bret R. Sellman
Bert L. Semler
Keun Seok Seo
Ruth Serra-Moreno
Peter Setlow
Mohammad R. Seyedsayamdost
Robert M. Q. Shanks
Lindsey N. Shaw
Lilach Sheiner
Aimee Shen
Bo Shopsin
Hanna E. Sidjabat
Lynn L. Silver
Jon T. Skare
Claudio Slamovits
Jason C. Slot
Nicolas Sluis-Cremer
Mark S. Smeltzer
Joe Smith
Thomas E. Smithgall
Erica Sonnenburg
Joseph A. Sorg
Maria João Sousa
Stephen Spatz
Joan Steitz
Dennis Stevens
Scott Stibitz
Christopher S. Sullivan
David J. Sullivan
Xingmin Sun
Carlos Pelleschi Taborda
Clifford C. Taggart
Andrew W. Tai
Rita Tamayo
Shumin Tan
Stefan Taube
Norbert Tautz
Timothy L. Tellinghuisen
David G. Thanassi
Casey Theriot
Vinai Chittezham Thomas
George Thompson
Ruth B. Thornton
David M. Tobin
Andrew Tomaras
Juan M. Tomas
Stanislas Tomavo
Chris J. Tonkin
Victor J. Torres
Lindsay Traeger
Frances Trail
Ana Traven
Cagla Tukel
Kürsad Turgay
Claire Elizabeth Turner
Nassos Typas
Antonio D. Uttaro
Marcelo A. Vallim
David VanDuin
Rob Van Houdt
Mark J. van Raaij
Elin Verbrugghe
Marco Vignuzzi
Pablo Vinuesa
Slavena Vylkova
Samuel Wagner
Irene Wagner-Doebler
Kevin John Waldron
David Walsh
David Wang
Qiuhong Wang
Yue Wang
Zhong Wang
Gary E. Ward
Matthew J. Wargo
Paula I. Watnick
Jeffrey L. Watts
Keith E. Weaver
Louis Weiss
Rachel Whitaker
Theodore C. White
Philipp Wiemann
John V. Williams
Craig Winstanley
Christiane E. Wobus
Alan J. Wolfe
Benjamin E. Wolfe
K. Eric Wommack
John L. Woolford
Karina B. Xavier
Jianping Xu
Jin-Rong Xu
Kyosuke Yamamoto
Shingo Yamamoto
Vincent B. Young
Sung-Hwan Yun
Robert Zagursky
Raffaele Zarrilli
Kai Zhang
Li Zhang

